# The Relationship of Reading Abilities With the Underlying Cognitive Skills of Math: A Dimensional Approach

**DOI:** 10.3389/fpsyg.2021.577488

**Published:** 2021-02-25

**Authors:** Luca Bernabini, Paola Bonifacci, Peter F. de Jong

**Affiliations:** ^1^Department of Psychology, University of Bologna, Bologna, Italy; ^2^Research Institute of Child Development and Education, University of Amsterdam, Amsterdam, Netherlands

**Keywords:** math, reading, working memory, approximate number system, phonological awareness

## Abstract

Math and reading are related, and math problems are often accompanied by problems in reading. In the present study, we used a dimensional approach and we aimed to assess the relationship of reading and math with the cognitive skills assumed to underlie the development of math. The sample included 97 children from 4th and 5th grades of a primary school. Children were administered measures of reading and math, non-verbal IQ, and various underlying cognitive abilities of math (counting, number sense, and number system knowledge). We also included measures of phonological awareness and working memory (WM). Two approaches were undertaken to elucidate the relations of the cognitive skills with math and reading. In the first approach, we examined the unique contributions of math and reading ability, as well as their interaction, to each cognitive ability. In the second approach, the cognitive abilities were taken to predict math and reading. Results from the first set of analyses showed specific effects of math on number sense and number system knowledge, whereas counting was affected by both math and reading. No math-by-reading interactions were observed. In contrast, for phonological awareness, an interaction of math and reading was found. Lower performing children on both math and reading performed disproportionately lower. Results with respect to the second approach confirmed the specific relation of counting, number sense, and number system knowledge to math and the relation of counting to reading but added that each math-related marker contributed independently to math. Following this approach, no unique effects of phonological awareness on math and reading were found. In all, the results show that math is specifically related to counting, number sense, and number system knowledge. The results also highlight what each approach can contribute to an understanding of the relations of the various cognitive correlates with reading and math.

## Introduction

The Triple Code Model (Dehaene, 1992, 1997) suggests that numbers are expressed in three different codes that are at the base of our ability to count and process numerosity. They have distinct functional neuroarchitectures and are related to performance on particular tasks (Van Harskamp and Cipolotti, 2001). The first one is a verbal code, connected to the linguistic system, that is used to recover well-learned arithmetic facts using memory, such as simple addition and multiplication tables (González and Kolers, 1982). The second one is a visual code that represents and spatially manages numbers in Arabic format (Ashcraft and Stazyk, 1981; Dahmen et al., 1982; Dehaene and Cohen, 1991; Weddell and Davidoff, 1991). Finally, the third code is the analog magnitude representation, which gives a representation of analogical quantity on a mental number line (approximate calculation and magnitude comparison) (Chochon et al., 1999; Spelke and Dehaene, 1999). According to this model, the verbal code is used in particular for counting, addition, and easy multiplication, while approximate calculation and comparison are sustained more by the non-verbal codes.

From a developmental perspective, some studies proposed that language is essential for the growth of numerical competencies (Hauser et al., 2010), and mathematical language was found to be a unique significant predictor of numeracy performance (Purpura and Logan, 2015). There is also evidence that the structure of the language system in which one grows up shapes the development of numerical concepts. For example, Chinese children have been found to have an advantage in arithmetic tasks because in Chinese the base 10 number system is transparently represented in the structure of the number words (Geary et al., 1996). On the counterpart, speakers of Mundurukù, who lack words for numbers beyond 5, are able to compare and add large approximate numbers, but they fail in exact arithmetic for large numbers (Pica et al., 2004). Others, however, argue that numerical competence, at least for some aspects, can develop independently from linguistic skills (Landerl et al., 2004). Landerl et al. (2004) support the theory that the number system is able to develop independently from the language domain. However, the relationship between linguistic and numerical skills is still under debate as well as the role of domain general cognitive markers as possible shared cognitive underpinning of reading and math skills. In the present study, we addressed the issue of the specificity of cognitive markers of math abilities and whether reading ability might also affect numerical competencies.

It is known that math and reading skills are related (Cirino et al., 2018; Koponen et al., 2020), and the co-occurrence of reading and math disorders can be between 2.3% and 40% (Lewis et al., 1994; Landerl and Moll, 2010; Moll et al., 2015; Koponen et al., 2018). In a recent meta-analysis, Joyner and Wagner (2020) reported that children with math disorders have a two times greater chance of having reading disability. According to the multiple deficit model (Pennington, 2006; McGrath et al., 2020), the relationship between math and reading can be accounted for by shared factors, which may act at different levels (genetics, cognitive, and behavioral).

A first candidate shared cognitive factor underlying reading and math is phonological processing, which might explain problems with the verbal code. Many studies have found that phonological processing difficulties predict early numeracy skills (Bonifacci et al., 2016) and the emergence of mathematical difficulties (Leather and Henry, 1994; Hecht et al., 2001; Rasmussen and Bisanz, 2005) and suggest that phonological awareness might be a shared underlying deficit of both disorders (Slot et al., 2016). Rapid automatized naming (RAN) is another important shared factor and is clearly distinct from phonological awareness (e.g., de Jong and van der Leij, 1999; Kirby et al., 2010). Naming speed has been found to explain a significant portion of the common variance of reading and math (Geary, 2011; Koponen et al., 2007, 2019, 2013; Fuchs et al., 2016; Balhinez and Shaul, 2019) and, together with timed counting, the largest amount of the overlap between the fluency of reading and math (Koponen et al., 2007, 2020). Also, domain-general processes, such as processing speed or working memory (WM), have been proposed to account for the relationship between math and reading as well as the comorbidity of math and reading problems (Bull and Johnston, 1997; Willcutt et al., 2013). A meta-analysis by Daucourt et al. (2020) suggested that domain-general risk factors underlie the co-occurrence of reading and math to a greater extent compared to the co-occurrence of reading and ADHD. Also, the genetic correlation between reading and math was higher than between reading and ADHD. In particular, there is evidence that a weakness in verbal WM leads to difficulties in storing and remembering arithmetic facts (Swanson and Sachse-Lee, 2001; Koponen et al., 2007, 2013; Simmons and Singleton, 2008; Vanbinst et al., 2015). Whereas some authors suggested that specific components of WM are related differentially to mathematics (Wilson and Swanson, 2001; Simmons et al., 2012), other studies note that the whole WM system is linked to mathematical knowledge development (Simmons et al., 2008; Zhang and Lin, 2015).

Two approaches have been used to enhance the understanding of the cognitive factors that underlie the common and specific aspects of reading and math. In one approach, underlying cognitive deficits of reading and math disorders are examined, with a particular emphasis on the shared and distinct markers of comorbid conditions and single deficits. In the other approach, underlying cognitive factors are used to predict common and unique variance in individual differences in reading and math in unselected samples. Hereafter, we will discuss main evidence deriving from the two approaches.

### Studies on Cognitive Deficits Underlying Reading and/or Math Disorders

The main aim of these studies is to examine the various deficits that are characteristic of the single- and comorbid-deficit groups. The results of these studies might have implications for the diagnosis and treatment of disorders in reading (RD) and math (MD). Of particular interest is whether the deficits of the comorbid group, MD + RD, can be characterized as an additive combination of the deficits found in the single MD and RD group. From a methodological point of view, the disorder conditions are dichotomous independent variables on which each deficit variable is regressed as a function of the cognitive skill and importantly both main effects of reading and math as well as their interaction are tested. The test of the interaction effect reveals whether the deficit in the comorbid group is an additive or non-additive combination of the deficits in the single-deficit groups.

The approximate number system (ANS) has been proposed as a specific deficit underlying math impairments (Butterworth and Laurillard, 2010; Piazza et al., 2010). ANS involves an automatic, non-symbolic, approximate sense of number that is available before the start of schooling, and that survives beyond the lifespan. Others propose a deficit in accessing numerosities from symbols (Noël and Rousselle, 2011; De Smedt et al., 2013). Schneider et al. (2017), in a meta-analysis, found that symbolic magnitude comparison skills were more strongly related to broader mathematical competence, e.g., counting, arithmetic, or algebra, compared to non-symbolic tasks. Within this view, symbolic numerical magnitude processing is thought to be as important to arithmetic development as phonological awareness is to reading (Vanbinst et al., 2016), as also documented by studies on children with math and reading disorder (MD-RD) (Landerl et al., 2004, 2009).

As said, a main issue is whether the two disorders and their comorbid phenotype might have distinct or common causes. Considering children with reading impairments, Simmons and Singleton (2008) hypothesized a weakness in the verbal code and, in particular, in recalling numerical facts. Indeed, several studies reported that children with dyslexia are slow in calculation, arithmetic fact retrieval, and, in particular, have difficulties with multiplication (Simmons and Singleton, 2006; Boets and De Smedt, 2010; De Smedt and Boets, 2010).

Most studies show that the MD + RD can be characterized by an additive combination of the deficits found in the single MD and RD group; this means that children with comorbid MD and RD usually show a summation of symptoms from the two disorders (e.g., phonological deficit and counting). van der Sluis et al. (2004) found that MD-only children were impaired in naming of digits and quantities, whereas RD-only children were impaired in digit and letter naming. The MD-only group also showed problems in executive functioning. The performance of the group with double deficits could best be described as an additive combination of the deficits underlying each disorder (see for similar results Willburger et al., 2008). Also, Landerl et al. (2009) found distinct cognitive profiles for RD and MD groups, with weaknesses in phonological awareness for the first, deficits in the processing of symbolic and non-symbolic magnitudes for the second, and additive cognitive deficits for the RD + MD group. Other studies (Cirino et al., 2007; Jordan et al., 2003) found that the RD-only group outperformed the RD + MD and MD-only group, with the latter groups showing a similar math profile. Finally, Moll et al. (2015) examined deficits in the underlying factors of reading and math. They found that factors underlying numerical difficulties in children with RD were different from the factors underlying numerical problems in children with MD. Children with RD were impaired in phoneme awareness and in RAN but not in simple reaction time (Bonifacci and Snowling, 2008). Furthermore, RD-only children performed more weakly on all tasks tapping verbal number skills, but they had no difficulty with either the non-symbolic number comparison or in locating numbers on the number line. Their weaknesses were particularly marked when numbers had to be transcoded. The MD group, instead, showed deficits in processing numerosities and in all math tasks. The cognitive profile of the RD + MD group did not differ from the single-deficit groups in mathematics and literacy skills but manifested a weaker performance than the RD group in some language measures (phonological awareness and verbal IQ). Importantly, none of the RD-by-MD interactions were significant, demonstrating again that the cognitive deficits of the comorbid group were simply the sum of the deficits of the single-disability group.

In summary, studies on cognitive deficits show that children with math problems have deficits in the processing of numerosity, both non-symbolic and symbolic, whereas children with a reading disorder tend to have deficits in those math-related abilities that require the use of the verbal code. Moreover, the deficits in the comorbid group were mainly found to be an additive effect of the deficits underlying each single disorder, suggesting that each disorder has specific markers that concur in comorbid conditions.

### Studies on Shared and Distinct Predictors of Reading and Math in Typical Populations

The main aim in the studies with unselected samples has been to examine the unique effects of a range of underlying cognitive abilities on reading and math. Shared abilities, having an effect on both reading and math, can account for their relation or overlap (e.g., McGrath et al., 2011; Koponen et al., 2020). Cognitive abilities that are specifically related to either math or reading are responsible for their differentiation. From a methodological point of view, all variables in these studies are usually considered to be continuous, and reading and math are simultaneously regressed on all the cognitive abilities.

A range of studies has focused on the shared and specific predictors of math and reading. In some of these studies, reading and math were specified as indicators of a common latent variable. In one of the first of this type of studies, Koponen et al. (2007) showed that letter knowledge and counting ability in kindergarten together with RAN in grade 4 predicted the common variance of reading and math fluency. This study did not include measures of number sense. In a more recent longitudinal study by the same group (see also Koponen et al., 2013, 2020), from first to second grade, the shared variance of reading and math fluency was almost fully explained by serial retrieval fluency, a latent variable which, in their structural equation model, was formed by RAN and counting. Also, phonological awareness, number comparison, and processing speed were predictors of shared reading and math fluency. Surprisingly, Koponen et al. (2020) did not find a specific relation of number sense, number comparison, and number writing with math. In an earlier longitudinal study from first to third grade, Fuchs et al. (2016) also observed that RAN had an effect on both reading and math skills, alongside with attentive behavior, reasoning, and visuospatial memory, albeit through retrieval measures. However, unlike Koponen et al. (2020), Fuchs et al. also found distinct predictors for reading (language, phonological memory, and RAN) and math (attentive behavior, reasoning, and WM). In a cross-sectional study with children from first to third grade, Balhinez and Shaul (2019) showed that the common predictors of reading and math might change over time. In particular, RAN was specific to math in first grade but predicted both reading and math fluency in later grades, whereas WM predicted both abilities in first and second grade but no longer in third grade. Finally, in a recent study, Vanbinst et al. (2020) examined the common and unique predictors of reading and math in kindergarten children. Interestingly, they included a range of cognitive abilities deemed to be specifically related to reading and math. Their results showed that non-symbolic and symbolic magnitude comparisons were unique predictors of math, whereas numeral recognition and phonological awareness were related to both reading and math. Similarly, Child et al. (2019) found in second grade children that numerosity, tested through a non-symbolic magnitude comparison task, was uniquely related to math, whereas phonological awareness and WM were related to both reading and math.

In summary, the studies adopting a continuous approach suggest a number of candidate shared predictors of reading and math, in particular phonological processing, RAN, counting, and WM. In contrast, some studies suggest that symbolic and non-symbolic number processing skills are unique predictors of math skills. However, most of these studies were conducted on young children, mainly from the end of the preschool to the first years of primary school. Little is known on the relations of the cognitive correlates of math with reading in older children who already mastered the first stages of reading and math acquisition.

#### Present Study

In this study, we aimed to assess the relationship of reading and math with the cognitive skills assumed to underlie the development of math. We hypothesized that tasks related to the number sense domain would be related only to math and not to reading. In addition, we expected that cognitive skills related to the phonological domain (phonological awareness) and domain-general abilities, in particular WM, would be related to both math and reading.

We used both approaches mentioned above to examine these relationships. In the cognitive deficit approach, our main question was whether the effects of math and reading on the various cognitive correlates were additive or, alternatively, whether the combination of skills in reading and math had additional positive or negative effects on the performance of the cognitive skills presumed to underlie the development of math.

Previous studies on the relations of math and reading with underlying cognitive skills have adopted a design with four groups: two single (MD and RD) and a double (MD + RD) deficit group and one group of typically developing children. The analysis of the data in such a design is straightforward: (multivariate) analysis of variance to examine the main effects of RD (yes or no) and MD (yes or no) and the MD-by-RD interaction effect. In principle, this means that each cognitive skill is regressed on three independent variables: the RD factor, the MD factor, and a factor for the RD-by-MD interaction. However, the deficit groups in such a design are the result of cut-offs on math and reading ability, which are generally considered as continuously distributed abilities. Such cut-offs are always somewhat arbitrary, and the outcomes of the study might be affected by the chosen cut-off (e.g., Landerl and Moll, 2010). Moreover, the use of extreme groups requires extensive screening, and it is therefore not very efficient. In this study, we adopted a continuous perspective, but following the approach in previous studies with various deficit groups, we examined the effects of reading and math skills as well as their interaction on the cognitive skills believed to underlie math development.

In the second approach, the cognitive abilities were used to predict math and reading ability. One question here was whether the various cognitive abilities contribute independently to individual differences in math and reading ability. A further question was whether the cognitive abilities are uniquely related to math and reading or related to what math and reading have in common.

The study was conducted with Italian fourth- and fifth-grade children. We administered measures of reading and arithmetic, non-verbal IQ, and various underlying cognitive abilities of arithmetic (counting, number sense, and number system knowledge). We also included measures of WM and phonological ability.

## Materials and Methods

### Participants

The sample consisted of 97 children (mean age = 9.8, *SD* = 0.6, 55.7% females), attending the 4th (57 children) and the 5th (40 children) grades of primary school, selected from five classes.

Participants were selected from schools in suburban areas in the north of Italy. From an initial sample of 126 children, we included in the study only participants with a complete dataset collected (29 children were excluded). All the remaining children met the following inclusion criteria: intellectual functioning within the normal range (>70 standard score), as measured through the matrix task of the Kaufman Brief Intelligence Test (KBIT-2, Kaufman and Kaufman, 2014; Bonifacci and Nori, 2016) and the absence of neurological impairment, sensory deficits, and neurodevelopmental disorders. Families were from a low to high socio-economic status (6.8% low, 23% medium-low, 43.2% medium, 23% medium-high, and 4% high), measured through the Hollingshead Four-Factor Index.

Parents provided written informed consent prior to the experiment. The Ethical Committee of the University of Bologna approved the study design.

### Measures

Children were administered tests assessing intellectual functioning, formal math skills, and reading tasks. A detailed description of the task is detailed below.

#### Non-verbal IQ

Children were administered the Matrices subtest of K-BIT 2 (Kaufman and Kaufman, 2014; Bonifacci and Nori, 2016). The test is a measure of non-verbal IQ. Depending on the age range, children were shown pictures (starting from one up to a matrix of 12 elements) and they were asked to choose among five to six images the one that best fitted with the target picture. For example, on top, there might be a picture of rain associated with an umbrella and the sun associated with a question mark and then pictures below that include gloves, socks, sunglasses, and shoes. The correct answer is that the sun goes with sunglasses. There are different starting points based on the participant’s age, and the task stops after four consecutive wrong responses. There are 46 items; a score of 1 is given for each correct answer and the maximum score is 46. Split-half reliability coefficient in developmental age (4–18 years) was 0.87.

#### Working Memory

Children were administered the digit span task (forward and backward) sequencing test (memory) of the subtest of the WISC-IV (Wechsler, 2003; Italian adaptation, Orsini et al., 2012). Children were required to repeat forward and backward series of numbers of increasing length. The task was stopped after two failures on a series of the same length. The score is the number of digits’ series that they can repeat correctly. The maximum score is 16 for the forward and 16 for the backward. The test–retest reliability was 0.79 for digits forwards and 0.74 for digits backward (Orsini et al., 2012). We added the scores of the forward and backward span into one score for WM.

#### Phonological Awareness

Children were administered the phonological processing, a subtest of the NEPSY-II battery (Korkman et al., 2007). Phonological processing is designed to assess phonological awareness through different tasks, with different starting points according to participants’ age. The task starts with syllables blending [Me-la → Mela (apple)], then with recognition of syllables within different words [e.g., which words contain the sound “aci” → “Bacio” (kiss)]. For age 9–11, tasks of elision of syllables within a word (say “stop” but without “p”) or by substituting one phoneme in a word with another (say “roba” with “s” instead of “b”) were administered. There are 53 items and the maximum score is 53. Reliability scores are not reported in the Italian test manual, but a good internal reliability (*r* > 0.80) and test–retest reliability = 0.78 were reported in the original manual (Brooks et al., 2009).

#### Mathematical Knowledge

The BDE-2 (Biancardi et al., 2016), developmental dyscalculia battery, was administered. The BDE-2 is composed of nine tests plus three optional tests (of which only “repetition of numbers” was administered) for the fourth and fifth primary classes. We performed Cronbach’s alpha and factorial analysis to test the internal consistency, and for the purpose of the present study, tasks were grouped in four main areas: counting, number sense knowledge, number system knowledge, and math.

#### Counting

In this task, the examiner asks the children to count aloud from 80 to 140 and records the time. Then, the experimenter asks the child to count backward from 140. The time given to do so is the time that the child needed to count forward from 80 up to 140. The score is the total of numbers the child said correctly backward within the allotted time.

#### Number Sense

This was evaluated using two different subtests: triplets and insertion. On the triplet task, children have 2 min to indicate on a paper record form the largest number in 18 sets of three numbers (e.g., 30,100, 31,000, and 30,009). The score is the total number of answers they give correctly in 2 min. The maximum score is 18. On the insertion task, children have 2 min to place a target number at the correct place in a series of three numbers arranged in ascending order. For example, they have to put on a paper record form the number 10 in the correct position between the numbers 5, 8, and 15. The number of items is 18. The score is the total number of correct items done in 2 min. The maximum score is 18. Cronbach’s alpha based on the two scales was 0.64.

#### Number System Knowledge

This task was evaluated using three different subtests: number reading, number writing, and repetition of number. In the number reading task children have 1 min to read aloud a list of numbers of increasing difficulty (three to six digits). The score is the total number of Digits they read correctly. Number writing and repetition give two scores. First, the child has to repeat the number (repetition of numbers), and then, the child has to write the number (number writing). There are 18 numbers, among which there are numbers with the 0 (e.g., 807 or 5,010) and numbers with 4, 5, and 6 digits (e.g., 27,463 or 346,879). A score of 1 is given for each number that the child repeats (repetition score) or writes (writing score) correctly. The maximum score for both scales is 18. Each of the three scores was converted to a *z*-score. Then, the three scores were added to obtain one score for number system knowledge. Cronbach’s alpha based on the three scales was 0.78.

#### Math and Reading Ability

Standard tests for math and reading fluency were administered.

#### Math

This task was evaluated using four subtests of the BDE-2 (Biancardi et al., 2016) referred to the calculation ability and speed: multiplication, mental calculation, quick calculation, and approximate calculation. Multiplication—the examiner reads 18 items in random order (e.g., 3 × 4, 7 × 9…). Children have 3 s to give an answer to each operation. The score is the total number of answers they give correctly within 3 s. The maximum score is 18. Mental calculation—the examiner reads 18 operations (nine additions and nine subtractions), and children have 30 s to answer each operation with the correct result. The score is the total of answers they give correctly. The maximum score is 18. Quick calculation—children have 2 min to write the correct results of as many mixed operations as possible (additions, subtractions, multiplications, and divisions) up to a maximum of 40. The score is the total of answers they give correctly in 2 min. Approximate calculation—children have 2 min to indicate the correct result of 18 operations, indicating it from the four options. For example, the operation is 75:5 and they have to choose between 80, 375, 15, and 5. The score is the total of answers they give correctly in 2 min. The maximum score is 18. Cronbach’s alpha of the sum score, calculated over the four tests, was 0.79.

#### Reading

The reading materials were two texts taken from the MT reading test, the Italian battery used to assess text reading speed and accuracy (Cornoldi et al., 2017). Children were required to read as fast and accurate as possible, and reading comprehension was not tested. The texts were different for the two different grades of primary school. The text used to assess children from the fourth grade of elementary school has 141 words, while that for children from the fifth grade has 236 words. For the purpose of the present study, we calculated reading fluency, that is, the number of words read aloud correctly in 1 min. Then, we compute the *z*-score within each grade using the reading fluency in order to have a unique score of this variable by grade. The test manual reports reliability coefficients between 0.75 and 0.87 for accuracy scores and between 0.94 and 0.97 for reading speed.

## Results

### Descriptive Statistics

We considered scores with a mean of more than 3.3 standard deviations from the grade mean as outliers. There were eight of such scores, three in fourth and five in fifth grade. Each outlier score belonged to a different child. There were three children with very low scores on number sense and two on number system knowledge. Three children had very high scores on math or reading fluency. All outliers were coded as missing.

Descriptive statistics for the children’s variables, separated by grade, are reported in Table 1.

**TABLE 1:** Descriptive statistics for grade 4 and grade 5.

		**Grade 4**				**Grade 5**			
	**Max**	**Mean**	**SD**	**Skew**	**Kurt**	**Mean**	**SD**	**Skew**	**Kurt**
Age (years)		9.52	0.48	0.07	−2.06	10.24	0.45	0.47	0.97
General cognitive ability (*n* correct items)	46	28.61	6.72	–0.03	0.50	31.48	6.63	–0.59	–0.62
Phonological awareness (*n* correct items)	53	46.44	3.19	–0.58	−0.01	47.65	3.28	–0.70	–0.28
Working memory (*n* correct items)	32	14.00	1.91	0.53	−0.26	15.03	2.79	0.31	–0.51
Counting	^*a*^	–0.27	1.69	0.26	0.81	0.38	2.02	–0.02	–0.47
Number sense	^*a*^	–0.07	1.37	–0.86	0.68	0.57	1.22	–1.04	0.68
Number system knowledge	^*a*^	–0.81	2.12	–0.53	0.05	1.57	1.97	–1.42	1.98
Math (*n* correct items)	94	50.17	12.21	0.80	1.71	67.97	14.45	–0.44	–1.00
Reading (words per minute)	141/236	85.34	21.16	0.62	−0.21	114.17	28.43	–0.27	0.45

*Means for reading fluency cannot be compared between grades because different (grade-appropriate) texts were used to assess words read per minute.*

*^*a*^*z*-scores over grades.*

Next, we computed the correlations among the variables. To control for grade, we computed within-grade standardized scores. Then, the eight missing scores, less than 1% of the total number of data points, were estimated using the EM method in SPSS. Correlations among the variables for the full sample, controlling for grade, are reported in Table 2.

**TABLE 2:** Pooled within-grade correlations among the variables.

**Measure**	**1**	**2**	**3**	**4**	**5**	**6**	**7**
General cognitive ability							
Phonological awareness	0.310**						
Working memory	0.229*	0.521**					
Counting	0.268**	0.431**	0.193				
Number sense	0.402**	0.269*	0.174	0.276**			
Number system knowledge	0.241*	0.547**	0.353**	0.545**	0.310**		
Math	0.322**	0.459**	0.183	0.622**	0.515**	0.629**	
Reading	0.149	0.304*	0.104	0.418**	0.193	0.315*	0.310*

*p < 0.01. **p < 0.001.

As expected, the correlation between phonological awareness and WM was substantial [*r*(95) = 0.521, *p* < 0.01]. Also, a high correlation was found between counting and number system knowledge [*r*(95) = 0.545, *p* < 0.01]. Of most interest were the correlations of the cognitive skills with math and reading. As expected, the relations of math with its underlying cognitive skills, counting, number sense, and number system knowledge, were highly significant [all *r*(95) > 0.5, *p* < 0.01). We found moderate relations of reading with counting [*r*(95) = 0.418, *p* < 0.01] and number system knowledge [*r*(95) = 0.315, *p* < 0.05], whereas its correlation with number sense was not significant.

### Prediction of Cognitive Abilities From Math and Reading

In this approach, we conducted regression analyses on the within-grade standardized scores to examine the unique contributions of arithmetic and reading ability, as well as their interaction, in the prediction of phonological awareness, WM, and the cognitive correlates of math. Note that in these analyses, reading, math, and the interaction of reading and math were the independent variables, although this does not imply that they act causally. In these analyses, we controlled for IQ. The results of the analyses are presented in Table 3.

**TABLE 3:** Results of the regression analyses predicting the cognitive abilities from math, reading, and the interaction of math and reading: standardized regression coefficients and *R*^2^.

**Predictor**	**PA**	**WM**	**Count**	**NS**	**NSK**
IQ	0.189*	0.195^+^	0.054	0.263**	0.041
Math	0.365**	0.116	0.520**	0.423**	0.582**
Reading	0.161^+^	0.038	0.249**	0.023	0.128
Math by reading	−0.176*	−0.073	0.076	0.000	−0.056
*R*^2^	0.297	−0.073	0.452	0.328	0.416

PA, phonological awareness; WM, working memory; Count, counting; NS, number sense; NSK, number system knowledge. *^+^p* < 0.10.

*p < 0.05. **p < 0.01.

**FIGURE 1:**
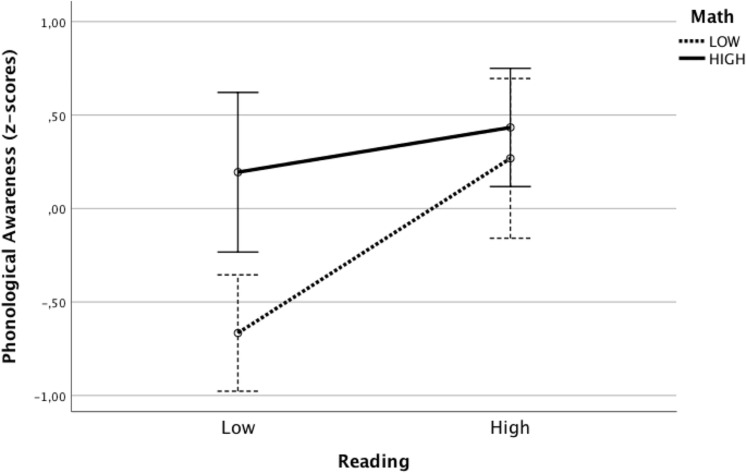
Interaction of math and reading for groups of lower and higher scoring children. Error bars: 95% confidence interval (CI).

We found an effect of math on phonological awareness. The effect of reading just missed significance (*p* = 0.085). We also found an interaction effect of math by reading. For a better understanding of the interaction effect, we formed groups of lower (score below the mean) and higher (score above the mean) performing children in math and reading. Cross classification of math (below or above average) and reading (below or above average) resulted in four groups. The mean scores of these groups are displayed in Figure 1.

The figure clearly shows that the lower performing children on both math and reading obtained a disproportionately lower score in phonological awareness.

Unexpectedly, we found no significant effects of reading or math on WM. Separate analyses for forward and backward memory span gave similar results.

The results with respect to the math-related cognitive skills were clear. Math was uniquely related to number sense and number system knowledge, whereas reading did not make a significant contribution. For counting, however, both math and reading made an independent contribution. The effect of math was about twice as large as the effect for reading. There were no math-by-reading interactions on the math-related cognitive skills.

### Prediction of Math and Reading by the Cognitive Abilities

In this approach, we also used the within-grade standardized scores, but now, we regressed math and reading ability on the cognitive abilities. In these analyses, we also controlled for IQ but omitted WM as we did not find any relationships with math and reading in the previous analyses. To examine the specific contributions of the cognitive abilities, we also conducted a set of regression analyses in which we controlled for reading ability in predicting math and for math in predicting reading. The results are reported in Table 4.

**TABLE 4:** Results of regression analyses predicting reading and math from underlying cognitive abilities: standardized regression coefficients and *R*^2^.

	**Math**	**Math**	**Reading**	**Reading**
Reading/math	–	−0.010	–	−0.020
IQ	0.017	0.017	−0.012	−0.012
PA	0.053	0.055	0.122	0.123
Count	0.337**	0.340**	0.320*	0.327*
NS	0.302**	0.302**	0.059	0.065
NSK	0.319**	0.320**	0.059	0.065
*R*^2^	0.595	0.596	0.199	0.199

PA, phonological awareness; Count, counting; NS, number sense; NSK, number system knowledge. *p < 0.05. **p < 0.01.

The main results of these analyses were that counting made a specific contribution to both reading and math. Number sense and number system knowledge were specifically related to math. In addition to our previous analysis in which each cognitive skill was regressed on reading and math (see Table 3), this approach revealed that counting, number sense, and number system knowledge made independent, that is, unique contributions to math. These analyses also show that phonological awareness did not describe independent variance in reading and math, although its correlation with both academic abilities was significant (see Table 2).

## Discussion

We examined the relationship of various proximal markers of math development with the common and unique aspects of math and reading. We also investigated the relations of math and reading with a domain-general ability, WM, and phonological awareness, a cognitive skill generally associated with reading development (e.g., Landerl et al., 2019). Unlike previous studies, we used two approaches to elucidate these relationships. The first is the deficit approach but here with math and reading as continuous predictors. Although in the present study we actually did not consider children with deficits, we kept the same term in continuity with previous research. In the other approach, regularly used in unselected samples, the cognitive abilities were taken to predict math and reading.

We considered counting, number sense, and number system knowledge as cognitive markers of math. As expected, all markers were moderately to highly related to math ability. Two math-related cognitive skills were also related to reading, that is, counting and number system knowledge, although their relationship with reading was lower than with math.

Next, we conducted two types of regression analyses. In the first type of analysis, the “deficit” approach, each cognitive skill was regressed on math and reading as well as their interaction. The outcomes denote the unique relations of math and reading with each math-related cognitive skill. We found here that the relation of number system knowledge with reading was no longer significant when math was included in the regression model. Thus, number system knowledge and number sense both had a specific relation with math, but not with reading. In contrast, counting had a unique relation with both reading and math. Finally, in the analyses on the cognitive markers of math, none of the math-by-reading interactions were significant. Thus, our continuous approach in this respect led essentially to the same results as studies that used a categorical approach, including groups that were weak in math, reading, or both (e.g., Moll et al., 2015).

In the second type of analysis, reading and math were regressed on the cognitive markers of math together with phonological awareness. The results here showed that all cognitive markers were independently related to math, even when reading was controlled. Counting was the only math-related skill that was also associated with reading. In all, both types of analyses clearly suggest that math is specifically related to counting, number sense, and number system knowledge. The second set of analyses adds here that each of these cognitive skills is independently related to math. Counting was found to be specifically related to both math and reading.

The specific relation of number sense to math seems understandable and aligns with previous findings in younger children (Child et al., 2019; Vanbinst et al., 2020). Following the Triple Code Model (Dehaene, 1992, 1997), number sense does not involve any verbal code and heavily taps numerosity and, in this study, particularly the representation of the number line. The finding seems in accordance with proposals to regard number sense as the prime characteristic of a math disorder (e.g., Piazza et al., 2010). Some authors suggested that number system knowledge might be a meaningful mediator of the relationship between approximate number system (ANS) skills and math competence (van Marle et al., 2014; Chu et al., 2015). Although it requires verbal skills, it might be cognitively conceptualized as a bridge function that, starting from basic ANS skills, allows to achieve higher order math competencies such as representing large quantities precisely and also facilitating the acquisition and storage of complex relations between numbers, more efficiently and precisely than does the ANS alone (Peng et al., 2017). However, note that other studies showed the selective contribution of transcoding to math performance over and above ANS skills (Göbel et al., 2014; Habermann et al., 2020). As expected, counting was specifically related to both math and reading. This finding is in line with the results reported in previous studies (e.g., Moll et al., 2015; Koponen et al., 2018). Counting requires, as reading, the activation of verbal labels and, as reading, is related to phonological awareness.

The results with respect to phonological awareness were less clear. As in previous studies, both math and reading, although the latter just missed significance, contributed to phonological awareness (Child et al., 2019). But, in this study, we found an interaction effect of math and reading. The effect of math became stronger when reading abilities decreased. In the group of relatively weak readers (below the mean in the sample), those who also had relatively low math skills had the worst performance in phonological awareness. Children with weak reading skills and good math skills had relatively spared phonological skills. These results are in line with previous findings that phonological processes might be important in some aspects of arithmetic skills and, particularly, for the comorbidity of math and reading disorders (Cirino et al., 2015; Slot et al., 2016). In the present study, phonological awareness might be viewed as a marker of the interaction of math and reading when these abilities are observed in a dimensional and continuous perspective. However, in the other type of regression analyses, phonological awareness did not contribute in the prediction of reading or math (see Table 4). Especially, the relation with math was fully captured by the math-specific cognitive markers, which also shared variance with phonological awareness. These results suggest that the role of phonological awareness may fade when stronger predictors of reading and math are considered in the regression model. Similarly, Koponen et al. (2020) found that the contribution of PA to the shared variance in reading and math was only very moderate; when RAN and counting were included in the prediction, they accounted for a higher amount of variance. Overall, however, it was striking that phonological awareness was hardly related to reading and even higher with math. A difference with earlier studies is that the current study involved older children. Especially in a transparent orthography like Italian, phonological awareness seems less relevant for reading in older children and thereby the relationship between these abilities might decrease (e.g., Landerl and Wimmer, 2000; de Jong and Van der Leij, 2003; Brizzolara et al., 2006). Another reason for the rather low relation between phonological awareness and reading could be that the measure of reading in this study concerned text reading and not the reading of a list of unrelated words. Finally, it might be that the phonological awareness task used was not sufficiently hard, as children’s performance was generally high (88% and 90% correct in grades 4 and 5, respectively), although not at ceiling, suggesting that there was relatively little variation on this task.

Somewhat to our surprise, we did not find relations of math and reading with WM. Also, relations were absent when memory span forward, usually more related to reading, and memory span backward, involving more executive functioning, were considered separately. It is not entirely clear why effects of reading and math on WM were not found. Possibly, the particular tasks used to assess math, mainly very simple calculations, and reading, texts, did not very heavily depend on WM. The absence of a relationship between WM and reading might be interpreted in the light of the debate as to whether phonological WM is a direct predictor of reading skills or, rather, involves access to representations that underlie phonological awareness tasks (Melby-Lervåg et al., 2012). Concerning math, many studies evidenced a primary role of the visuo-spatial WM component (Simmons et al., 2012; Zhang and Lin, 2015), and therefore, verbal WM might play a minor role. Furthermore, WM tasks and domain-general factors seem to be more strongly related to complex math outcomes such as problem solving tasks (Swanson and Beebe-Frankenberger, 2004; Fuchs et al., 2008, 2010) and procedural computations (Fuchs et al., 2010). These results are in line with previous evidence suggesting that domain-general skills might act indirectly via more proximal predictors (Cirino et al., 2018; Zoccolotti et al., 2020).

The present study has some limitations that could be addressed in future investigations. First, a larger sample size would have strengthened the generalizability of the findings. More specific limitations are referred to the tasks adopted in the study. The task used to assess ANS skills are not standard ANS tasks as they involve, at least in part, transcoding skills and number ordering. Symbolic order processing is related to a certain degree to number sense (magnitude processing) but does not completely overlap with it (Lyons et al., 2014; Sasanguie et al., 2017; Sasanguie and Vos, 2018). We also have to acknowledge that we did not include rapid automatized naming (RAN), which is known to be an important common predictor of reading and math. However, our main interest was in the relation of reading ability to the cognitive markers of math as derived from the Triple Code Model. There is already an abundant number of studies to show the relation of RAN to both math and reading. Finally, although we tested regression models in order to understand different patterns of predictors, we cannot infer causal relationships; longitudinal studies would be necessary at this regard.

In sum, we used two approaches to examine the relationship of reading ability with the main cognitive markers of math. The main findings were that predicting each cognitive marker from reading and math ability, we found that number sense and number system knowledge were specifically related to math, whereas counting was related to both math and reading. There were no math-by-reading interactions. In the second approach, all markers of math were used simultaneously to predict math and reading, respectively. The results confirmed the previous results on the relations of the various markers to reading and math, but these analyses also showed that counting, number sense, and number system knowledge independently contributed to individual differences in math.

A potential implication of this study for research is that the “deficit” approach can be adopted with the use of continuous indicators of individual differences in math and reading. The approach is fully compatible with the use of deficit groups, and the results of the present study seem to be in line with those of previous studies focusing on children with math and/or reading disorders. Moreover, from an educational perspective, a deficit approach might sometimes be too strict. Children with low reading skills, although not in the clinical range, might encounter subtle weaknesses also in the math domain and the other way around. A more comprehensive awareness of shared mechanisms underlying learning skills would allow to better promote scholastic well-being and achievements.

## Data Availability Statement

The raw data supporting the conclusions of this article will be made available by the authors, without undue reservation.

## Ethics Statement

The studies involving human participants were reviewed and approved by Bioethics Commmitte, University of Bologna. Written informed consent to participate in this study was provided by the participants’ legal guardian/next of kin.

## Author Contributions

LB carried out the study, collected and analyzed data, and wrote a preliminary version of the manuscript. PJ designed and supervised manuscript structure. PJ and LB performed statistical analysis. PJ and PB revised and significantly contributed to the final version of the manuscript. All authors discussed the results and contributed to the final manuscript.

## Conflict of Interest

The authors declare that the research was conducted in the absence of any commercial or financial relationships that could be construed as a potential conflict of interest.

## References

[B1] Ashcraft M. H. Stazyk E. H. (1981). Menatal addition: a test of three verification models. *Mem. Cogn.* 9 185–196. 10.3758/BF03202334 7242333

[B2] Balhinez R. Shaul S. (2019). The relationship between reading fluency and arithmetic fact fluency and their shared cognitive skills: a developmental perspective. *Front. Psychol.* 10:1281. 10.3389/fpsyg.2019.01281 31214086 PMC6555082

[B3] Biancardi A. Bachmann C. Nicoletti C. (2016). *BDE 2 - Batteria Discalculia Evolutiva.* Portland, OR: Erickson.

[B4] Boets B. De Smedt B. (2010). Single-digit arithmetic in children with dyslexia. *Dyslexia* 16 183–191. 10.1002/dys.403 20440746

[B5] Bonifacci P. Nori R. (2016). *KBIT-2. Kaufman Brief Intelligence Test Second Edition. Contributo alla Taratura Italiana [Contribution to Italian Standardization].* Firenze: Giunti-OS.

[B6] Bonifacci P. Snowling M. J. (2008). Speed of processing and reading disability: a cross-linguistic investigation of dyslexia and borderline intellectual functioning. *Cognition* 107 999–1017. 10.1016/j.cognition.2007.12.006 18272144

[B7] Bonifacci P. Tobia V. Bernabini L. Marzocchi G. M. (2016). Early literacy and numeracy skills in bilingual minority children: toward a relative independence of linguistic and numerical processing. *Front. Psychol.* 7:1020. 10.3389/fpsyg.2016.01020 27458413 PMC4935724

[B8] Brizzolara D. Chilosi A. Cipriani P. Di Filippo G. Gasperini F. Mazzotti S. (2006). Do phonologic and rapid automatized naming deficits differentially affect dyslexic children with and without a history of language delay? A study of Italian dyslexic children. *Cogn. Behav. Neurol.* 19 141–149. 10.1097/01.wnn.0000213902.59827.19 16957492

[B9] Brooks B. L. Sherman E. M. Strauss E. (2009). NEPSY-II: a developmental neuropsychological assessment. *Child Neuropsychol.* 16 80–101.

[B10] Bull R. Johnston R. S. (1997). Children’s arithmetical difficulties: contributions from processing speed, item identification, and short-term memory. *J. Exp. Child Psychol.* 65 1–24. 10.1006/jecp.1996.2358 9126630

[B11] Butterworth B. Laurillard D. (2010). Low numeracy and dyscalculia: identification and intervention. *ZDM Int. J. Math. Educ.* 42 527–539. 10.1007/s11858-010-0267-4

[B12] Child A. E. Cirino P. T. Fletcher J. M. Willcutt E. G. Fuchs L. S. (2019). A cognitive dimensional approach to understanding shared and unique contributions to reading, math, and attention skills. *J. Learn. Disabil.* 52 15–30. 10.1177/0022219418775115 29779434 PMC6212329

[B13] Chochon F. Cohen L. Van De Moortele P. F. Dehaene S. (1999). Differential contributions of the left and right inferior parietal lobules to number processing. *J. Cogn. Neurosci.* 11 617–630. 10.1162/089892999563689 10601743

[B14] Chu F. W. Vanmarle K. Geary D. C. (2015). Early numerical foundations of young children’s mathematical development. *J. Exp. Child Psychol.* 132 205–212. 10.1016/j.jecp.2015.01.006 25705049

[B15] Cirino P. T. Child A. E. Macdonald K. T. (2018). Longitudinal predictors of the overlap between reading and math skills. *Contemp. Educ. Psychol.* 54 99–111. 10.1016/j.cedpsych.2018.06.002 30559576 PMC6294126

[B16] Cirino P. T. Fletcher J. M. Ewing-Cobbs L. Barnes M. A. Fuchs L. S. (2007). Cognitive arithmetic differences in learning difficulty groups and the role of behavioral inattention. *Learn. Disabil. Res. Pract.* 22 25–35. 10.1111/j.1540-5826.2007.00228.x

[B17] Cirino P. T. Fuchs L. S. Elias J. T. Powell S. R. Schumacher R. F. (2015). Cognitive and mathematical profiles for different forms of learning difficulties. *J. Learn. Disabil.* 48 156–175. 10.1177/0022219413494239 23851137 PMC4065636

[B18] Cornoldi C. Colpo G. Carretti B. (2017). *Prove MT - Kit Scuola.* Florence: Giunti Edu.

[B19] Dahmen W. Hartje W. Büssing A. Sturm W. (1982). Disorders of calculation in aphasic patients- Spatial and verbal components. *Neuropsychologia* 20 145–153. 10.1016/0028-3932(82)90004-5 6178056

[B20] Daucourt M. A. Erbeli F. Little C. W. Haughbrook R. Hart S. A. (2020). A meta-analytical review of the genetic and environmental correlations between reading and attention-deficit/hyperactivity disorder symptoms and reading and math. *Sci. Stud. Read.* 24 23–56. 10.1080/10888438.2019.1631827 32189961 PMC7079676

[B21] de Jong P. F. van der Leij A. (1999). Specific contributions of phonological abilities to early reading acquisition: results from a Dutch latent variable longitudinal study. *J. Educ. Psychol.* 91 450–476. 10.1037/0022-0663.91.3.450

[B22] de Jong P. F. Van der Leij A. (2003). Developmental changes in the manifestation of a phonological deficit in dyslexic children learning to read a regular orthography. *J. Educ. Psychol.* 95:22. 10.1037/0022-0663.95.1.22

[B23] De Smedt B. Boets B. (2010). Phonological processing and arithmetic fact retrieval: evidence from developmental dyslexia. *Neuropsychologia* 48 3973–3981. 10.1016/j.neuropsychologia.2010.10.018 20965205

[B24] De Smedt B. Noël M. P. Gilmore C. Ansari D. (2013). How do symbolic and nonsymbolic numerical magnitude processing skills relate to individual differences in children’s mathematical skills? A review of evidence from brain and behavior. *Trends Neurosci. Educ.* 2 48–55. 10.1016/j.tine.2013.06.001

[B25] Dehaene S. (1992). Varieties of numerical abilities. *Cognition* 44 1–42. 10.1016/0010-0277(92)90049-N 1511583

[B26] Dehaene S. (1997). “Babies who count,” in *The Number Sense: How the Mind Creates Mathematics*, ed. Dehaene S., (Oxford: Oxford University Press), 41–63.

[B27] Dehaene S. Cohen L. (1991). Two mental calculation systems: a case study of severe acalculia with preserved approximation. *Neuropsychologia* 29 1045–1074. 10.1016/0028-3932(91)90076-K 1723179

[B28] Fuchs L. S. Fuchs D. Stuebing K. Fletcher J. M. Hamlett C. L. Lambert W. (2008). Problem solving and computational skill: are they shared or distinct aspects of mathematical cognition? *J. Educ. Psychol.* 100:30. 10.1037/0022-0663.100.1.30 20057912 PMC2802329

[B29] Fuchs L. S. Geary D. C. Compton D. L. Fuchs D. Hamlett C. L. Seethaler P. M. (2010). Do different types of school mathematics development depend on different constellations of numerical versus general cognitive abilities? *Dev. Psychol.* 46:1731. 10.1037/a0020662 20822213 PMC2976828

[B30] Fuchs L. S. Geary D. C. Fuchs D. Compton D. L. Hamlett C. L. (2016). Pathways to third-grade calculation versus word-reading competence: are they more alike or different? *Child Dev.* 87 558–567. 10.1111/cdev.12474 26700885 PMC4809764

[B31] Geary D. C. (2011). Cognitive predictors of achievement growth in mathematics: a 5-year longitudinal study. *Dev. Psychol.* 47:1539. 10.1037/a0025510 21942667 PMC3210883

[B32] Geary D. C. Bow-Thomas C. C. Liu F. Siegler R. S. (1996). Development of arithmetical competencies in Chinese and American children: influence of age, language, and schooling. *Child Dev.* 67 2022–2044. 10.2307/1131607 9022227

[B33] Göbel S. M. Watson S. E. Lervåg A. Hulme C. (2014). Children’s arithmetic development: it is number knowledge, not the approximate number sense, that counts. *Psychol. Sci.* 25 789–798. 10.1177/0956797613516471 24482406

[B34] González E. G. Kolers P. A. (1982). Mental manipulation of arithmetic symbols. *J. Exp. Psychol. Learn. Mem. Cogn.* 8 308–319. 10.1037/0278-7393.8.4.308

[B35] Habermann S. Donlan C. Göbel S. M. Hulme C. (2020). The critical role of Arabic numeral knowledge as a longitudinal predictor of arithmetic development. *J. Exp. Child* 193:104794. 10.1016/j.jecp.2019.104794 32062163

[B36] Hauser M. Chomsky N. Fitch W. (2010). “The faculty of language: what is it, who has it, and how did it evolve?,” in *The Evolution of Human Language: Biolinguistic Perspectives (Approaches to the Evolution of Language*, eds Larson R. Déprez V. Yamakido H., (Cambridge, MA: Cambridge University Press), 14–42.

[B37] Hecht S. A. Torgesen J. K. Wagner R. K. Rashotte C. A. (2001). The relations between phonological processing abilities and emerging individual differences in mathematical computation skills: a longitudinal study from second to fifth grades. *J. Exp. Child Psychol.* 79 192–227. 10.1006/jecp.2000.2586 11343408

[B38] Jordan N. C. Hanich L. B. Kaplan D. (2003). A longitudinal study of mathematical competencies in children with specific mathematics difficulties versus children with comorbid mathematics and reading difficulties. *Child Dev.* 74 834–850. 10.1111/1467-8624.00571 12795393 PMC2791887

[B39] Joyner R. E. Wagner R. K. (2020). Co-occurrence of reading disabilities and math disabilities: a meta-analysis. *Sci. Stud. Read.* 24 14–22. 10.1080/10888438.2019.1593420 32051676 PMC7015531

[B40] Kaufman A. S. Kaufman N. L. (2014). *Kaufman Brief Intelligence Test, Second Edition.* London: Pearson Assessment.

[B41] Kirby J. R. Georgiou G. K. Martinussen R. Parrila R. (2010). Naming speed and reading: from prediction to instruction. *Read. Res. Q.* 45 341–362. 10.1598/RRQ.45.3.4

[B42] Koponen T. Aunola K. Ahonen T. Nurmi J. E. (2007). Cognitive predictors of single-digit and procedural calculation skills and their covariation with reading skill. *J. Exp. Child Psychol.* 97 220–241. 10.1016/j.jecp.2007.03.001 17560969

[B43] Koponen T. Aunola K. Nurmi J. E. (2019). Verbal counting skill predicts later math performance and difficulties in middle school. *Contemp. Educ. Psychol.* 59:101803. 10.1016/j.cedpsych.2019.101803

[B44] Koponen T. Eklund K. Heikkilä R. Salminen J. Fuchs L. Fuchs D. (2020). Cognitive correlates of the covariance in reading and arithmetic fluency: importance of serial retrieval fluency. *Child Dev.* 91 1063–1080. 10.1111/cdev.13287 31292957

[B45] Koponen T. Salmi P. Eklund K. Aro T. (2013). Counting and RAN: predictors of arithmetic calculation and reading fluency. *J. Educ. Psychol.* 105:162. 10.1037/a0029285

[B46] Koponen T. K. Sorvo R. Dowker A. Räikkönen E. Viholainen H. Aro M. (2018). Does multi-component strategy training improve calculation fluency among poor performing elementary school children? *Front. Psychol.* 9:1187. 10.3389/fpsyg.2018.01187 30050486 PMC6050482

[B47] Korkman M. Kirk U. Kemp S. (2007). NEPSY-Second Edition (NEPSY-II). *J. Psychoeduc. Assess.* 28 175–182. 10.1177/0734282909346716

[B48] Landerl K. Bevan A. Butterworth B. (2004). Developmental dyscalculia and basic numerical capacities: a study of 8-9-year-old students. *Cognition* 93 99–125. 10.1016/j.cognition.2003.11.004 15147931

[B49] Landerl K. Freudenthaler H. H. Heene M. De Jong P. F. Desrochers A. Manolitsis G. (2019). Phonological awareness and rapid automatized naming as longitudinal predictors of reading in five alphabetic orthographies with varying degrees of consistency. *Sci. Stud. Read.* 23 220–234. 10.1080/10888438.2018.1510936

[B50] Landerl K. Fussenegger B. Moll K. Willburger E. (2009). Dyslexia and dyscalculia: two learning disorders with different cognitive profiles. *J. Exp. Child Psychol.* 103 309–324. 10.1016/j.jecp.2009.03.006 19398112

[B51] Landerl K. Moll K. (2010). Comorbidity of learning disorders: prevalence and familial transmission. *J. Child Psychol. Psychiatry* 51 287–294. 10.1111/j.1469-7610.2009.02164.x 19788550

[B52] Landerl K. Wimmer H. (2000). Deficits in phoneme segmentation are not the core problem of dyslexia: evidence from German and English children. *Appl. Psycholinguist.* 21 243–262. 10.1017/S0142716400002058

[B53] Leather C. V. Henry L. A. (1994). Working memory span and phonological awareness tasks as predictors of early reading ability. *J. Exp. Child Psychol.* 58 88–111. 10.1006/jecp.1994.1027 8064220

[B54] Lewis C. Hitch G. J. Walker P. (1994). The prevalence of specific arithmetic difficulties and specific reading difficulties in 9- to 10-year-old boys and girls. *J. Child Psychol. Psychiatry* 35 283–292. 10.1111/j.1469-7610.1994.tb01162.x 8188799

[B55] Lyons I. M. Price G. R. Vaessen A. Blomert L. Ansari D. (2014). Numerical predictors of arithmetic success in grades 1-6. *Dev. Sci.* 17 714–726. 10.1111/desc.12152 24581004

[B56] McGrath L. M. Pennington B. F. Shanahan M. A. Santerre-Lemmon L. E. Barnard H. D. Willcutt E. G. (2011). A multiple deficit model of reading disability and attention-deficit/hyperactivity disorder: searching for shared cognitive deficits. *J. Child Psychol. Psychiatry* 52, 547–557. 10.1111/j.1469-7610.2010.02346.x 21126246 PMC3079018

[B57] McGrath L. M. Peterson R. L. Pennington B. F. (2020). The multiple deficit model: progress, problems, and prospects. *Sci. Stud. Read.* 24 7–13. 10.1080/10888438.2019.1706180 32440085 PMC7241589

[B58] Melby-Lervåg M. Lyster S. A. H. Hulme C. (2012). Phonological skills and their role in learning to read: a meta-analytic review. *Psychol. Bull.* 138 322–352. 10.1037/a0026744 22250824

[B59] Moll K. Göbel S. M. Snowling M. J. (2015). Basic number processing in children with specific learning disorders: comorbidity of reading and mathematics disorders. *Child Neuropsychol.* 21 399–417. 10.1080/09297049.2014.899570 24697279

[B60] Noël M.-P. Rousselle L. (2011). Developmental changes in the profiles of dyscalculia: an explanation based on a double exact-and-approximate number representation model. *Front. Hum. Neurosci.* 5:165. 10.3389/fnhum.2011.00165 22203797 PMC3243900

[B61] Orsini A. Pezzuti L. Picone L. (2012). *WISC-IV.* Firenze: Giunti-OS.

[B62] Peng P. Yang X. Meng X. (2017). The relation between approximate number system and early arithmetic: the mediation role of numerical knowledge. *J. Exp. Child Psychol.* 157 111–124. 10.1016/j.jecp.2016.12.011 28142096

[B63] Pennington B. F. (2006). From single to multiple deficit models of developmental disorders. *Cognition* 101 385–413. 10.1016/j.cognition.2006.04.008 16844106

[B64] Piazza M. Facoetti A. Trussardi A. N. Berteletti I. Conte S. Lucangeli D. (2010). Developmental trajectory of number acuity reveals a severe impairment in developmental dyscalculia. *Cognition* 116 33–41. 10.1016/j.cognition.2010.03.012 20381023

[B65] Pica P. Lemer C. Izard V. Dehaene S. (2004). Exact and approximate arithmetic in an Amazonian indigene group. *Science* 306 499–503. 10.1126/science.1102085 15486303

[B66] Purpura D. J. Logan J. A. R. (2015). The nonlinear relations of the approximate number system and mathematical language to early mathematics development. *Dev. Psychol.* 51 1717–1724. 10.1037/dev0000055 26436871

[B67] Rasmussen C. Bisanz J. (2005). Representation and working memory in early arithmetic. *J. Exp. Child Psychol.* 91 137–157. 10.1016/j.jecp.2005.01.004 15890174

[B68] Sasanguie D. Lyons I. M. De Smedt B. Reynvoet B. (2017). Unpacking symbolic number comparison and its relation with arithmetic in adults. *Cognition* 165 26–38. 10.1016/j.cognition.2017.04.007 28460351

[B69] Sasanguie D. Vos H. (2018). About why there is a shift from cardinal to ordinal processing in the association with arithmetic between first and second grade. *Dev. Sci.* 21:e12653. 10.1111/desc.12653 29417697

[B70] Schneider M. Beeres K. Coban L. Merz S. Susan Schmidt S. Stricker J. (2017). Associations of nonsymbolic and symbolic numerical magnitude processing with mathematical competence: a meta-analysis. *Dev. Sci.* 20:e12372. 10.1111/desc.12372 26768176

[B71] Simmons F. Singleton C. Horne J. (2008). Brief report–Phonological awareness and visual-spatial sketchpad functioning predict early arithmetic attainment: evidence from a longitudinal study. *Eur. J. Cogn. Psychol.* 20 711–722. 10.1080/09541440701614922

[B72] Simmons F. R. Singleton C. (2006). The mental and written arithmetic abilities of adults with dyslexia. *Dyslexia* 12 96–114. 10.1002/dys.312 16734354

[B73] Simmons F. R. Singleton C. (2008). Do weak phonological representations impact on arithmetic development? A review of research into arithmetic and dyslexia. *Dyslexia* 14 77–94. 10.1002/dys.341 17659647

[B74] Simmons F. R. Willis C. Adams A. (2012). Different components of working memory have different relationships with different mathematical skills. *J. Exp. Child Psychol.* 111 139–155. 10.1016/j.jecp.2011.08.011 22018889

[B75] Slot E. M. van Viersen S. de Bree E. H. Kroesbergen E. H. (2016). Shared and unique risk factors underlying mathematical disability and reading and spelling disability. *Front. Psychol.* 7:803. 10.3389/fpsyg.2016.00803 27375508 PMC4901067

[B76] Spelke E. Dehaene S. (1999). Biological foundations of numerical thinking: response to T.J. Simon (1999). *Trends Cogn. Sci.* 3 365–366. 10.1016/S1364-6613(99)01385-6 10498925

[B77] Swanson H. L. Beebe-Frankenberger M. (2004). The relationship between working memory and mathematical problem solving in children at risk and not at risk for serious math difficulties. *J. Educ. Psychol.* 96 471–491. 10.1037/0022-0663.96.3.471

[B78] Swanson H. L. Sachse-Lee C. (2001). A subgroup analysis of working memory in children with reading disabilities: domain-general or domain-specific deficiency? *J. Learn. Disabil.* 34 249–263. 10.1177/002221940103400305 15499879

[B79] van der Sluis S. de Jong P. F. van der Leij A. (2004). Inhibition and shifting in children with learning deficits in arithmetic and reading. *J. Exp. Child Psychol.* 87 239–266. 10.1016/j.jecp.2003.12.002 14972600

[B80] Van Harskamp N. J. Cipolotti L. (2001). Selective impairments for addition, subtraction and multiplication. Implications for the organisation of arithmetical facts. *Cortex* 37 363–388. 10.1016/S0010-9452(08)70579-3 11485063

[B81] van Marle K. Chu F. W. Li Y. Geary D. C. (2014). Acuity of the approximate number system and preschoolers’ quantitative development. *Dev. Sci.* 17, 492–505. 10.1111/desc.12143 24498980

[B82] Vanbinst K. Ansari D. Ghesquière P. De Smedt B. (2016). Symbolic numerical magnitude processing is as important to arithmetic as phonological awareness is to reading. *PLoS One* 11:e0151045. 10.1371/journal.pone.0151045 26942935 PMC4778857

[B83] Vanbinst K. Ceulemans E. Ghesquière P. De Smedt B. (2015). Profiles of children’s arithmetic fact development: a model-based clustering approach. *J. Exp. Child Psychol.* 133 29–46. 10.1016/j.jecp.2015.01.003 25731679

[B84] Vanbinst K. van Bergen E. Ghesquière P. De Smedt B. (2020). Cross-domain associations of key cognitive correlates of early reading and early arithmetic in 5-year-olds. *Early Childhood Res. Q.* 51 144–152. 10.1016/j.ecresq.2019.10.009

[B85] Wechsler D. (2003). *WISC-IV Administration Manual. The Wechsler Intelligence Scale for Children—Fourth Edition.* London: Pearson Assessment.

[B86] Weddell R. A. Davidoff J. B. (1991). A dyscalculic patient with selectively impaired processing of the numbers 7, 9, and 0. *Brain Cogn.* 17 240–271. 10.1016/0278-2626(91)90076-K 1799453

[B87] Willburger E. Fussenegger B. Moll K. Wood G. Landerl K. (2008). Naming speed in dyslexia and dyscalculia. *Learn. Individ. Diff.* 18 224–236. 10.1016/j.lindif.2008.01.003

[B88] Willcutt E. G. Petrill S. A. Wu S. Boada R. DeFries J. C. Olson R. K. (2013). Comorbidity between reading disability and math disability: concurrent psychopathology, functional impairment, and neuropsychological functioning. *J. Learn. Disabil.* 46 500–516. 10.1177/0022219413477476 23449727 PMC3749272

[B89] Wilson K. M. Swanson H. L. (2001). Are mathematics disabilities due to a domain-general or a domain-specific working memory deficit? *J. Learn. Disabil.* 34 237–248. 10.1177/002221940103400304 15499878

[B90] Zhang X. Lin D. (2015). Pathways to arithmetic: the role of visual-spatial and language skills in written arithmetic, arithmetic word problems, and nonsymbolic arithmetic. *Contemp. Educ. Psychol.* 41 188–197. 10.1016/j.cedpsych.2015.01.005

[B91] Zoccolotti P. De Luca M. Marinelli C. V. Spinelli D. (2020). Predicting individual differences in reading, spelling and maths in a sample of typically developing children: a study in the perspective of comorbidity. *PLoS One* 15:e0231937. 10.1371/journal.pone.0231937 32352985 PMC7192483

